# Magnetic resonance findings of neurofibromatosis type 2: a case report

**DOI:** 10.4076/1757-1626-2-6720

**Published:** 2009-07-02

**Authors:** Gabriela Spilberg, Edson Marchiori, Emerson L Gasparetto, Rafael Ferracini Cabral, Tatiana Chinem Takayassu, Raquel Ribeiro Batista, Isabela Garcia Vieira

**Affiliations:** 1 Department of Radiology of the Federal University of Rio de Janeiro, Rua Professor Rodolpho Paulo Rocco, 255, Cidade Universitária, CEP 21941-913, Rio de Janeiro, Brazil; 2 Department of Radiology of the Fluminense Federal University, Rua Marquês do Paraná, 530, Centro, CEP 24000-000 Niterói, Rio de Janeiro, Brazil

## Abstract

Neurofibromatosis type 2 is an inherited autosomal dominant syndrome, characterized by multiple neoplasms of the central and peripheral nervous system associated with ocular abnormalities. The most common tumor associated with the disease is the vestibulocochlear schwannoma (VIII cranial nerve), and as many as 10% of patients with this tumor have neurofibromatosis type 2. In this report we aim to present a 34-year-old male who was seen for bilateral hearing loss. During his workup, which included cranial computer tomography, he was found to have multiple intracranial masses. Cranial and whole spine magnetic resonance imaging showed bilateral vestibulocochlear schwannoma, multiple meningiomas, and one intramedullary tumor. Based on clinical and imaging findings the diagnostic of neurofibromatosis type 2 was made.

## Introduction

Neurofibromatosis type 2 is a rare disease, with incidence of 1 in 33,000 to 40,000 [1,2]. Male and female patients are approximately equally affected [3]. Until 1987 neurofibromatosis type 1 and 2 were considered one single disease, but it was then demonstrated that the two disorders arose from different mutations in different chromosomes [4]. NF2 is an inheritable disorder with an autosomal dominant mode of transmission, associated with chromosome 22q12 [5]. It is suspected that approximately one-half of cases are inherited, and one-half are the result of new, de novo mutations [6].

The aim of this report is to present a case of sporadic NF2, in which diagnosis was established based on medical history, clinical symptoms and image findings on magnetic resonance (MR) imaging.

## Case presentation

A 34-year-old Caucasian Brazilian male was seen at an outside facility for progressive bilateral hearing loss. A head computed tomography (CT) showed multiple intracranial masses. He was then referred to our institution for further work up. Physical examination revealed an alert and oriented patient, with normal cognitive function. He had bilateral hearing loss and diminished visual acuity. Few scattered skin lesions that resembled skin tags were also found. The clinical history included a past of tonic-clonic seizures, treated with fenitoin for a year. Also, a complaint of chronic headache associated with vertigo was identified.

He was referred for an otologic evaluation, which included an audiometric exam that showed bilateral sensoneural hearing loss, profound on the right, and moderate on the left. Also, the brainstem evoked response audiometry showed absence of response on the right and only one wave on the left.

**Figure 1:**
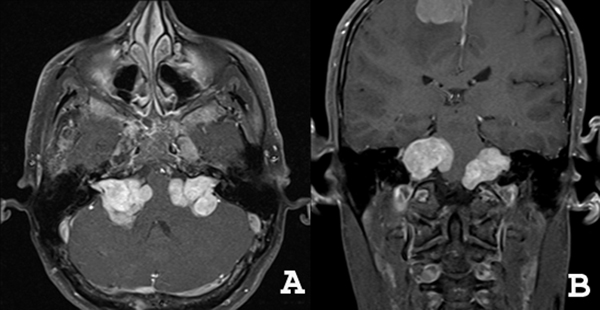
**Vestibular schwannomas**. Axial **(A)** and coronal **(B)** enhanced T1-weighted MR images demonstrating bilateral solid masses in the cerebellopontine angles, compressing the pons and the 4th ventricle. In addition, a right parafalcine meningioma is seen.

**Figure 2:**
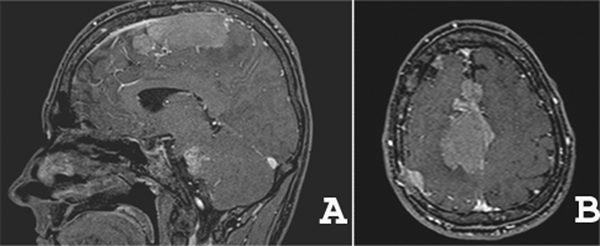
**Sagittal (A) and axial (B) T1 weighted contrast enhanced MR images showing multiple dural based homogeneous enhancing masses, suggesting the diagnosis hypothesis of multiple meningiomas**.

**Figure 3:**
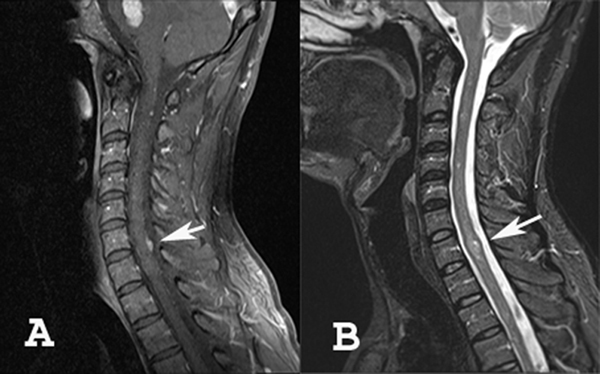
**(A) sagittal T1 contrast-enhanced MR image of the cervical spine**. A dural-based enhancing mass in seen at the C6-C7 level (arrow), suggestive of meningioma. **(B)** sagittal T2-weighted MR image of the spine. A focal hyperintense abnormality is demonstrated at the C7 level (arrow), compatible with intramedullary tumor (ependymoma or astrocytoma).

Review of the brain MR imaging findings showed bilateral acoustic schwannoma, and another four enhancing masses consistent with meningiomas, involving the parafalcine region, sphenoid wing, frontal and parietal lobes (Figures 1 and 2). Spine MR imaging findings included one cervical intramedullary tumor, and one cervical spine meningioma (Figure 3). He was then referred to Neurosurgery Department for surgical treatment.

Although he had no family history, the image findings of bilateral acoustic schwannoma, multiple intracranial meningiomas, and one intramedullary tumor, suggested the diagnosis of NF2.

## Discussion

The term MISME has been proposed to the NF 2 syndrome, due to multiple inherited schwannomas (MIS), meningiomas (M) and ependymomas (E). In addition to the neoplasms, posterior subcapsular lenticular opacity (juvenile cortical cataract) is often present. Patients with NF2 may have cutaneous schwannomas that resemble skin tags, but they rarely have café -au-lait spots and do not demonstrate the cutaneous neurofibromas like NF1. In 1997 Gutmann et al. proposed revised criteria for diagnostic of the syndrome [7] (Table 1).

**Table 1:** [Gutmann et al ([7])]

**Definite diagnoses of NF2**
• Bilateral CN VIII schwannomas on MRI or CT scan (no biopsy necessary)
• First-degree relative with NF2 and either unilateral early-onset CN VIII schwannoma (age <30 y) or any two of the following:
• Meningioma
• Glioma
• Schwannoma
• Juvenile posterior subcapsular lenticular opacity (juvenile cortical cataract)
**Presumptive diagnoses of NF2**
• Early onset of unilateral CN VIII schwannomas on MRI or CT scan detected in patients younger than 30 years and one of the following:
• Meningioma
• Glioma
• Schwannoma
• Juvenile posterior subcapsular lenticular opacity
• Multiple meningiomas (>2) and unilateral CN VIII schwannoma or one of the following:
• Glioma
• Schwannoma
• Juvenile posterior subcapsular lenticular opacity

The diagnosis of NF2 is usually made in the second or third decade of life, with a peak in the 20s. The clinical presentation of NF2 varies, but approximately a range from 30-45% of patients are diagnosed because of symptoms resulting from cranial nerve (CN) VIII schwannomas, such as hearing loss, tinnitus, balance impairment, and weakness in CN VII distribution. The reason for this is that CN VIII schwannomas are symptomatic at a relatively small size. The tumor causes symptoms by compressing or stretching the cochlear nerve, compressing the blood supply to the nerve or to the cochlea, or causing hemorrhage into the nerve or cochlea [8]. In Mautner et al [9] series bilateral CN VIII schwannomas were found in 43 patients (90%), and unilateral CN VIII schwannomas were found in 3 (6%).

Several authors have studied series of cases to try to define incidence of the tumors in cases of NF2. Mautner et al studied 48 patients with NF2, in which the prevalence of findings were: vestibular schwannomas (CN VIII) in 46 (96%), spinal tumors in 43 (90%), posterior subcapsular cataracts in 30 (63%), meningiomas in 28 (58%), and trigeminal schwannomas in 14 (29%) [9]. Aoki et al.[10] reported cranial MR of 11 patients. In their series, all patients had acoustic schwannomas, 8 had other cranial nerve tumors (5 multiple and 3 single) and 6 had meningiomas (4 multiple and 2 single).

The majority of intramedullary spine tumors in NF2 is ependymomas and arises in either the upper cervical cord or the conus. Meningiomas present as intradural extramedullary neoplasms that are very similar to spontaneous meningiomas and most commonly involve the thoracic spine; frequently they are multiple in number. Patronas et al [11] studied a series of 49 patients with NF2 with spinal MR images, which demonstrated spinal cord and/or canal tumors in 31 (63%). Twenty-six patients (53%) had intramedullary lesions, 27 patients (55%) had intradural extramedullary tumors, and 22 patients (45%) had at least one tumor of each type. Our patient had bilateral CN VIII schwannomas, five meningiomas, four intracranial and one spinal, and one intramedullary tumor, with no histopathological diagnosis. The schwannomas were the cause of his bilateral hearing loss. The parafalcine meningioma had invaded and thrombosed his superior sagittal sinus, probably the cause of his headaches and vertigo. Although he had no family history, finding of bilateral schwannomas is diagnostic for the syndrome, without the need of a biopsy.

## Abbreviations

CN: Cranial nerve; CT: Computed tomography; MISME: Multiple inherited schwannomas, meningiomas and ependymomas; MR: Magnetic resonance; NF2: Neurofibromatosis type 2.

## Consent

Written informed consent was obtained from the patient for publication of this case report and any accompanying images. A copy of the written consent is available for review by the Editor-in-Chief of this journal.

## Competing interests

The authors declare that they have no competing interests.

## Authors' contributions

GS conceived the study. RFC, TCT, IGV and RRB performed the literature review. GS, ELG, and EM edit and coordinated the manuscript. All authors read and approved the final manuscript.
